# Sport participation, acculturative stress, and depressive symptoms among international college students in the United States

**DOI:** 10.3389/fpsyt.2023.1104325

**Published:** 2023-03-02

**Authors:** Hyosoon Yim, Amy Chan Hyung Kim, James Du, Jeffrey D. James

**Affiliations:** Department of Sport Management, Center for Sport, Health, and Equitable Development, Florida State University, Tallahassee, FL, United States

**Keywords:** sport participation, acculturative stress, depressive symptoms, international student, acculturation, mental health

## Abstract

**Introduction:**

The distinctive social nature of sport in its capacity to promote immigrants’ adaptation to the new society has been regarded as a vehicle to cope with adverse mental health outcomes derived from acculturative stress (AS) and feelings of marginalization. However, the evidence on the relationship between sport participation (SP), AS, and mental health have been lacking and fragmented. Recognizing this challenge, we examined the mediating effect of AS on the relationship between SP and depressive symptoms (DS) among international college students in the USA.

**Methods:**

A total of 203 international college students in the USA were recruited *via* Prolific. The instrumentation included previously validated measures: SP (SLIM-18), AS (ASSIS), DS (CES-D-10), sense of coherence (SOC-13), and demographic characteristics.

**Results:**

Mediation analysis showed a significant association between (1) SP and DS (ß = −0.030, *p* < 0.05) and (2) AS and DS (ß = 0.053, *p* < 0.001), while (3) no significant indirect effect of AS was found [ß = −0.001, SE = 0.0003, 95% CI (−0.008, 0.004)].

**Discussion:**

Even though several previous scholars have argued that SP is an effective tool to cope with AS among international students, the present study implies this may not be applied to all international students. Specifically, AS and DS among European participants were lower than those from non-European countries, including Asia. Future studies using meta-analysis could be beneficial to examine the external validity of the previous studies on the relationship between SP, acculturation, and mental health to address this potential heterogeneity on the level of AS based on their origin countries or continents. The current study provides meaningful implications for adopting the transformative marketing perspective, which is a marketing approach that pursues positive social outcomes by promoting positive behavior of the target population.

## 1. Introduction

The number of international students in the USA has increased exponentially, marked by a 119% expansion over the past two decades (1). Considering not only their contribution to the USA economy [i.e., $28.4 billion in 2021; (2)] but also the significant diversity and value they add to the educational environment, many university administrators and service units are putting their effort to improve international college students’ experience inside and outside of their campus (3). Undoubtedly, the prevalence of adverse mental outcomes among this population has been one of the most critical challenges. For example, in a screening test for depressive symptoms (DS) held by Rice et al. (4), 36.7% of the international students either exceeded or reached the clinical cut-off limit. Similar evidence was found by Han et al. (5), who pointed out that 45% of Chinese international students at a large American university reported symptoms of depression. Even after recognizing the universal preponderance curve of DS in the USA (6) and worldwide (7), it should be particularly noted that international college students are more vulnerable to DS than their domestic peers (8).

International students’ unique stressors fuel the spread of various concerns, resulting in a relatively higher rate of DS (9–11). Such a phenomenon should be with considering acculturation, a vital process they go through in a new society. Acculturation is a dual reciprocal process of cultural and psychological change resulting from contact between two or more cultural groups and their members (12). During the acculturation process, immigrants face various challenges due to the different demands of the new society. As one type of immigrant, it is inevitable for international students to undergo the same process (13). The psychological challenges experienced through the acculturation process are highly dependent on acculturative stress (AS). AS refers to unique stress caused by the adaptation process of immigrants, such as homesickness or perceived discrimination (14). It buffers international students’ adaption, exerting ongoing long-term effects on their DS (4, 15–17).

Physical activity (PA) has been regarded as a promising precaution to cope with DS for the general public (18, 19). A similar result was also found among international college students. Han et al. (5) found a negative association between the amount of exercise and the levels of anxiety and DS among Chinese students. As one dominant form of PA, sport participation (SP) has extraordinary potential as a coping strategy for DS. Compared to unorganized exercise (e.g., weightlifting) or leisure (e.g., kayaking), sport has a unique social nature (20), where individuals polish their physical prowess to achieve competitive results, being engaged in a social relationship (e.g., teammates, competitors, coaches). Due to such nature, SP’s psychological [e.g., DS, subjective wellbeing, happiness; (21–23)] and social benefits [e.g., social capital, social interaction; (21, 24, 25)] have been identified in the various populations including general public (22), adolescents (21) and older adults (23).

When it comes to the studies of SP, AS, and DS among international students, we identified two major research gaps. First, there is a lack of empirical evidence to confirm the impact of SP on AS among international college students. Even though several scholars investigated the relationship between SP and acculturation, the conceptualization of sport in these studies broadly embraced unorganized PA (26), exercise (27, 28), or leisure (29), indicating the lack of information on the role of organized SP. Further, the aforementioned scholars investigated the acculturation of general immigrants. Among the few empirical studies that explored the relationship between SP and AS among international students, Zhou et al. (30) and Lee et al. (31) found that participating in sport-oriented leisure can be a way to help international students cope with the stress derived from the unique challenges of acculturation. However, both studies adopted in-depth interviews with a small number of participants, and generalizability remained unclear. Second, previous scholars have overlooked the mediating role of AS in the relationship between SP and DS.

**FIGURE 1:**
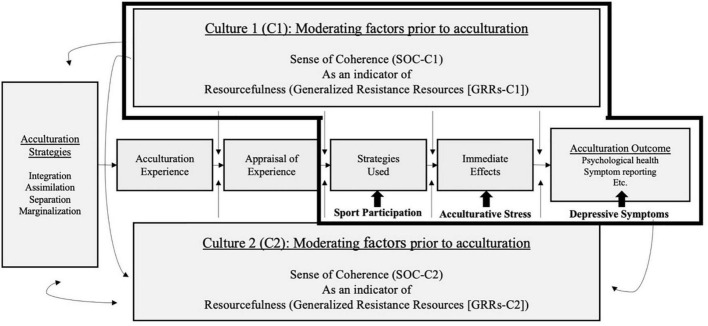
Integrative framework of acculturation and salutogenesis (32).

To fill these gaps, we aimed to investigate (1) the relationship between organized SP, AS, and DS and (2) the mediating effect of AS on the relationship between the level of SP and DS among international college students. The integrative framework of acculturation and salutogenesis (32) was adopted as a theoretical framework. According to Riedel et al. (32), the following steps generate an immigrant’s mental health outcome. First, facing unfamiliar environments and social norms (Step 1; acculturation experience), individuals evaluate the perceived difficulty (Step 2; appraisal of experience) and determine their coping strategies (Step 3; strategy used). The adopted strategies influence immediate stress levels in the next step (Step 4; immediate effect). The transition between each step mentioned above is moderated by various salutary factors conceptualized as generalized resistance resources [GRRs; personal and environmental factors that fosters capability for resilience or resistance throughout life experiences; (33)]. The GRRs include internally (e.g., constitution, competencies, and personal traits) and externally (e.g., social support, social stratum, and cultural stability) accumulated positive resources for dealing with potential challenges. Riedel et al. (32) suggested that the GRRs can be captured by one’s sense of coherence [SOC; one’s capability of comprehensibility, manageability, and meaningfulness obtained by his or her previous culture; (33)]. Finally, their long-term mental health outcome is determined by synthesizing the outcomes from the aforementioned steps. The mental outcome formulates a new sense of coherence, and the process is reproduced repeatedly. For the present study, we aimed to capture international students’ mental outcomes focusing on the process starting from the “strategy used” step (see Figure 1).

**FIGURE 2:**
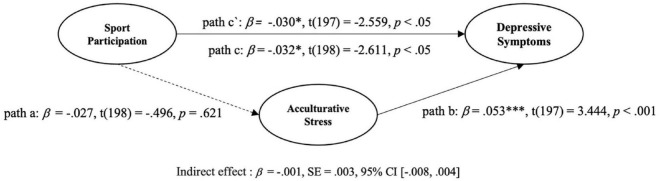
Mediation analysis of acculturative stress (AS) on sport participation (SP) and depressive symptoms (DS). *p < 0.05, **p < 0.01, ***p < 0.001.

Taken together, the following hypotheses were established (see Figure 2):

Hypothesis 1: One’s level of SP will be negatively associated with AS.

Hypothesis 2: One’s level of SP will be negatively associated with DS.

Hypothesis 3: One’s level of AS will be positively associated with DS.

Hypothesis 4: Acculturative stress mediates the negative relationship between SP and DS.

## 2. Materials and methods

### 2.1. Participants and study design

Respondents were recruited *via* Prolific, a crowdsourcing platform where individuals complete paid tasks for various organizations. Prolific has been selected for the present study considering its advantages in which investigators could obtain a more diverse sample (34). The survey was conducted from 27 April 2020 to 18 May 2020. After obtaining consent, respondents were directed to Qualtrics to confirm their international student status. We also included one attention question (i.e., Please check “Completely Agree” for this question). Among the 211 recorded responses that passed the screening questions, three responses that failed to pass the attention question were excluded. Additionally, five outliers were excluded based on the Mahalanobis distance (35), Cook’s distance (36), and leverage point (37). In sum, a total of 203 questionnaires were included for analysis.

### 2.2. Measures

#### 2.2.1. Sport participation

Self-reported SP levels were assessed using an abbreviated version of the serious leisure inventory measure [SLIM; (38)], a validated measurement tool for sport-related leisure used in various populations (CFI = 0.91; *a* = 0.97), including international students (31). The questionnaire included 18 items to record the seriousness of sport-related leisure activity as well as perceived personal and social rewards as a result of the participation (38). To accurately measure organized SP, excluding unorganized leisure (e.g., dog walking), the respondents were guided to “fill in the blank” with their favorite sports, such as soccer, tennis, or golf, as they read and responded to each item. Additionally, we made sure to estimate not spectating but participation by adding “playing” or “I play” to some of the original items (e.g., from “_ for me is an expression of myself” to “Playing _ for me is an expression of myself”). A 9-point Likert scale was used to measure each item (1 = Completely Disagree to 9 = Completely Agree) and higher scores represented higher levels of SP.

#### 2.2.2. Acculturative stress

We adopted the AS scale for international students [ASSIS; (39)] to measure AS. The scale consists of four sub-constructs (i.e., perceived discrimination, homesickness, perceived hate, fear, stress due to the change, guilt, and miscellaneous). Each of the constructs was measured with two to 10 items (36 items in total) on a five-point scale ranging from 1 = Completely Disagree to 5 = Completely Agree and higher scores representing higher levels of AS. ASSIS was specifically designed and validated for international students (40), hence considered a better option than other similar instruments developed for general immigrants [e.g., the SAFE scale; (41)].

#### 2.2.3. Depressive symptoms

The Center for Epidemiologic Studies Depression Scale [CES-D-10; (42)] was utilized to estimate an individual’s DS. As an abbreviated version of the CES-D (43), the scale has been frequently utilized and validated in research targeting a wide range of age groups, including the college student population (44). The items reflect the feelings of the respondents (e.g., “I was bothered by things that usually do not bother me”; “I felt depressed”), and each item ranged from 0 = Rarely (< 1 day per week) to 3 = Mostly (5–7 days per week). A higher score indicates more DS among the participants.

#### 2.2.4. Covariates

A total of nine items were included for measuring the sociodemographic characteristics of age, race, gender, home country, time stayed in the USA, average sport playing time, current academic program, TOEFL score, and amount of funding. Based on the previous findings, time stayed in the USA [i.e., length of residence; (16, 45–47)], TOEFL score [i.e., language barrier; (48–50)], and amount of funding [i.e., financial status; (51–53)] was deemed as influential factors of AS or DS, hence, controlled as covariates. Additionally, the sense of coherence from previous culture [SOC-C1 one’s capability of comprehensibility, manageability, and meaningfulness obtained by his or her previous culture; (33)] was included as a covariate. The SOC-13 has three subscales which are comprehensibility (5 items), manageability (4 items), and meaningfulness (4 items) using a 7-point Likert scale [1 = Never (0% chance) to 7 = Every time (100% chance)]. Higher scores indicate robust SOC. To estimate SOC from the previous culture, we modified each item into past tense by adding “Before you came to the US” (e.g., from “How often do you have the feeling that you are not sure you can keep under control?” to “Before you came to the US, how often do you have the feeling that you are not sure you can keep under control?”).

### 2.3. Analyses

Regression analysis was employed to estimate path coefficients. Path *a* connects the predictor variable SP and the mediating variable AS; path *b* links the mediating variable AS and the outcome variable DS; and path *c‘* links the predictor variable SP with the outcome DS considering the mediating variable AS whereas path *c* presents the total effect of the predictor variable SP on the outcome variable DS (see Figure 2). The indirect effect was assessed using Hayes’s bootstrapping approach using the PROCESS macro-Version 3.3 (54). This technique uses 5,000 bootstrap samples for bias correction to set the 95% confidence intervals. Each path was deemed significant if the *p*-value was smaller than 0.05 and the bootstrap confidence interval did not include zero (54).

## 3. Results

Table 1 displays descriptive statistics of respondents. Roughly 69% of respondents were male, and most participants were 20’s (72.41%), Caucasian (58.10%) from European countries (70.9%). Approximately 37.9% of the students stayed in the USA for more than one and < 2 years, followed by < 1 year (30%). Including 20.7% of native English speakers, more than half (58.1%) of the respondents’ TOEFL scores indicated an advanced English level (55). Finally, while the majority had funding (73.4%), < $ 5,000 was the most common range of financial support among the respondents.

**TABLE 1:** Descriptive statistics of sample demographic profile.

	*n*	%	Cumulative %
**Age (Years) (*M* = 23.43; SD = 4.62)**			
18–19	37	18.23	18.23
20–29	147	72.41	90.64
30–39	17	8.37	99.01
40 and over	2	0.99	100.0
**Race**			
Caucasian	118	58.1	58.1
Black	9	4.4	62.6
Hispanic	30	14.8	77.3
Asian	19	9.4	86.7
Other	27	13.3	100.0
**Gender**			
Male	140	69.0	69.0
Female	63	31.0	100.0
**Home country (continent)**			
Africa	9	4.4	4.4
Asia	14	6.9	11.3
Europe	144	70.9	82.3
North America	23	11.3	93.6
Oceania	3	1.5	95.1
South America	10	4.9	100.0
**Time stayed in the USA (years) (*M* = 20.97; SD = 22.34)**			
< 1	61	30.05	30.05
1 ≤ *n* < 2	78	38.42	68.47
2 ≤ *n* < 3	37	18.23	86.7
3 ≤ *n* < 4	16	7.88	94.58
4 and over	11	5.42	100
**Time Playing Sports (hours/week) (*M* = 6.12; SD = 4.22)**			
0	2	0.99	0.99
1–4	91	44.83	45.81
5–9	70	34.48	80.3
10–14	30	14.78	95.07
15–19	5	2.46	97.54
20 and over	5	2.46	100
**TOEFL score**			
0–79	6	3.0	3.0
80–89	22	10.8	13.8
90–99	57	28.1	41.9
100–109	61	30.0	71.9
110–120	15	7.4	79.3
Waived	42	20.7	100.0
**Amount of funding/scholarship ($ per year)**			
0	54	26.6	26.6
1∼$ 5,000	85	41.9	68.5
5,001∼$ 10,000	35	17.2	85.7
10,001∼$ 15,000	18	8.9	94.6
15,001∼$ 20,000	7	3.4	98.0
20,001∼$ 25,000	3	1.5	99.5
Above $ 25,000	1	0.5	100.0

Table 2 presents descriptive statistics and Pearson correlation coefficients of the study variables. SP was not significantly associated with AS (*r* = −0.340, *p* = 0.632) but significantly negatively associated with DS (*r* = −0.175, *p* < 0.05). Additionally, AS was had a significant negative association with DS (*r* = 0.408, *p* < 0.01).

**TABLE 2:** Descriptive statistics and Pearson correlation coefficients of study variables.

Variable	1	2	3	*M* (SD)
1. SP	1	−0.340	−0.175*	6.55 (1.27)
2. AS		1	0.408**	2.42 (0.69)
3. DS			1	1.18 (0.56)

SP, sport participation; AS, acculturative stress; DS, depressive symptoms. *p < 0.05, **p < 0.01.

Figure 2 displays the results of mediation analyses of AS on SP and DS. The results showed that SP significantly predicted DS [*R*^2^ = 0.387, F(6, 196) = 20.662, *p* < 0.05; H2 supported] and AS (*p* < 0.001; H3 supported). However, because AS was not significantly predicted by SP [*R*^2^ = 0.169, F(5, 197) = 8.036, *p* = 0.621; H1 not supported], the mediation effect was also not significant [ß = −0.001, SE = 0.0003, 95% CI (−0.008, 0.004); H4 not supported].

## 4. Discussion

The primary aim of the current study was to explore the relationship between SP, AS, and DS among international students in the USA while considering the mediating effect of AS on the relationship between SP and DS. The association between SP and AS was insignificant (H1 not supported). While several previous scholars have argued that SP can be an effective tool for coping with AS among international students (31), the present study implies that this may not apply to all international students. We suspect that a relatively low level of AS among the current sample may be the potential reason. According to the results of descriptive analysis, the average level of the participants’ perceived AS (*M* = 2.42, SD = 0.69) in the present study was relatively low. Specifically, the average response regarding AS items was close to “Disagree” (i.e., 2.42 out of 5), indicating the respondents did not perceive their migration-related challenges severely. We suggest future researchers investigate whether a significant association between SP and AS exist among international college students with higher AS.

Acculturative stress was significantly and positively associated with DS (H2 supported). This result supports the work of previous scholars (40, 46, 56, 57), who suggested a higher level of AS predicts a significant increase in psychological distress. Yet, it is notable that the correlation rho between AS and DS in the present study (*r* = 0.053) was much smaller than that of the previous study investigated a similar number of international students [e.g., (16); *r* = 0.053]. Such a difference likely stems from a disparate sample characteristic of the two studies. While Zhang (16) investigated Chinese international college students from a single university campus, we examined students from diverse countries attending various universities. Previous scholars (49, 58) have pointed out that Asian international students in the USA had higher levels of AS than students from other continents. In the current study, Asian respondents’ average level of AS (*M* = 2.74, SD = 0.58) was higher than that of students from other continents (*M* = 2.40, SD = 0.70). The result obtained from the Mann–Whitney U test also revealed the significant difference of AS level between Asian and non-Asian group (*U* = 1,768.00, *p* < 0.05).

On the other hand, a comparison of descriptive statistics between European and non-European participants indicated that European respondents reported a relatively lower level of AS (*M* = 2.30, SD = 0.64) than their counterparts from non-European countries (*M* = 2.71, SD = 0.73) and this difference was statistically significant (*U* = 4,537.00, *p* < 0.001). Considering European respondents represented 70.9% of the current sample, we cautiously predict that their relatively low AS level may contribute to the low correlation between AS and DS. Future studies employing meta-analysis could be beneficial to examine the external validity of the previous studies on the relationship between SP, AS, and DS to address this potential heterogeneity on the level of AS based on their origin countries or continents.

The findings also indicated that SP among international college students significantly and negatively predicted DS (H3 supported). This result supports previous scholars’ findings regarding positive psychological outcomes of organized SP (21–23, 59, 60) and extends it to the international student population.

Finally, the mediating effect of AS between SP and DS was not significant (H4 not supported) due to the insignificant association between SP and AS. We included participants’ sense of coherence, length of residency, English fluency, and financial status as covariates, but there may be other major variables that may significantly influence one’s AS and DS. For instance, social support has been regarded as one of the influential factors regarding international students’ AS and DS (49, 61). Academic perfectionism (17, 46), and sport teammates [e.g., international-only-team versus mixed team; (62, 63)] are also potential covariates future scholars should consider controlling. Sport teammates, especially, can potentially be crucial covariate considering the unique social nature of SP. The authors did not control this factor due to the existing controversy regarding whether it fosters immigrants’ acculturation (63) or reinforce their ethnic identity (62) and partically buffers acculturation process.

The distinctive social nature of sport in its capacity to promote immigrants’ adaptation to the new society represents a promising remedy to cope with adverse mental health outcomes (30, 31). Our empirical findings provide meaningful implications for further strategic plans regarding universities’ health agenda. Specifically, by developing and implementing an organized sport delivery system, university administrators could foster their international students’ social bonds in the campus community. Also, from a transformative marketing perspective [i.e., a marketing approach that pursues positive social outcomes by promoting positive behavior of the target population; (64)], SP promotion should be implemented as a strategy at the institution, state, and national levels. Further, considering the significance of government- and policy-level influence on population health (65), future studies may need to investigate the effectiveness of SP on one’s mental health from a social-ecological perspective (66).

### 4.1. Limitations and future implications

The current study retained some limitations that have implications for future research. First, our convenience sample of international students included more European students (*n* = 144; 70.9%) while obtaining relatively few respondents from Africa (*n* = 9; 4.4%), Asia (*n* = 14; 6.9%), and Oceania (*n* = 3; 1.5%). Especially, only five students from China and India [the two biggest “sender” countries; (67)] participated in the survey. Accordingly, our results should not be generalized to whole international student populations. Second, considering previous findings indicating distinct effects that various types of sport exert on mental health (68), future studies may explore the effects of SP on mental health among international college students according to the different types of sport categories. Finally, the data collection was conducted during the early stage of the COVID-19 pandemic (i.e., from 27 April 2020 to 18 May 2020). Considering the widespread fears and strict regulations regarding sport activity in this period, it is reasonable to expect that international students would not be likely to play sports as usual. Since society is now heading to the “new normal,” and various alternative formats of SP (e.g., sport training using smartphone apps) have emerged, it would be timely to revisit the positive impact of SP.

## Data availability statement

The raw data supporting the conclusions of this article will be made available by the authors, without undue reservation.

## Ethics statement

This studies involving human participants were reviewed and approved by the Office for Human Subjects Projection, Florida State University. The patients/participants provided their written informed consent to participate in this study.

## Author contributions

HY, AK, JD, and JJ contributed to the conception and design of the study. HY organized and performed the statistical analyses under AK’s supervision. All authors contributed to manuscript revision, read, and approved the submitted version.
